# Intrapapillary hemorrhage with concurrent peripapillary and vitreous hemorrhage in two healthy young patients

**DOI:** 10.1186/s12886-018-0833-z

**Published:** 2018-07-13

**Authors:** In Hee Moon, Sung Chul Lee, Min Kim

**Affiliations:** Department of Ophthalmology, Institute of Vision Research, Severance Hospital, Yonsei University College of Medicine, 211, Eonjuro, Gangnam-gu, Seoul, Republic of Korea 135-270

**Keywords:** Intrapapillary hemorrhage, Myopia, Optic disc, Peripapillary subretinal hemorrhage

## Abstract

**Background:**

Cases of intrapapillary hemorrhage with adjacent peripapillary subretinal hemorrhage usually appear in myopic eyes with tilted optic discs, can improve without any specific treatment, and very rarely recur. But there has been no report of the use of advanced multimodal imaging such as spectral domain optical coherence tomography. We describe two rare cases of intrapapillary hemorrhage with adjacent peripapillary subretinal hemorrhage in an 11-year-old female and a 16-year-old male.

**Case presentation:**

An 11-year-old female with no past history presented with floaters in her right eye. Her BCVA (best-corrected visual acuity) by the Snellen was 20/20. Fundus examination, optical coherence tomography (OCT) revealed intrapapillary hemorrhage, peripapillary subretinal hemorrhage. After 3 weeks, all hemorrhage was resolved. Similarly, a 16-year-old male with no past history presented with blurry vision, black filamentous floaters. His BCVA was 20/20 in both eyes. Fundus examination showed intrapapillary hemorrhage, peripapillary subretinal hemorrhage while OCT revealed peripapillary edema in his right eye. Multimodal imaging did not reveal any presence of optic disc drusen. After 4 weeks of observation, the hemorrhage resolved. Cases of intrapapillary hemorrhage with peripapillary subretinal hemorrhage have rarely been reported.

**Conclusions:**

This condition generally affects monocularly, in myopic eyes with tilted discs. Despite an unknown cause, the hemorrhages spontaneously resolved without any treatment. Consistent with the good visual prognosis reported previously, the vision, optic nerve function of the two patients were preserved. It should be differentiated from other causes of subretinal hemorrhage.

## Background

Cases of intrapapillary hemorrhage with adjacent peripapillary subretinal hemorrhage have been rarely reported. [1, 2] Patients often present with sudden floater symptoms, blurry vision, scotoma, and nonspecific decreases in visual acuity. The cases usually appear in myopic eyes with tilted optic discs, can improve without any specific treatment, and very rarely recur. [1, 2] Cases of intrapapillary hemorrhage with adjacent peripapillary subretinal hemorrhage have been reported only once in Korea [3], but there has been no report of the use of advanced multimodal imaging such as spectral domain optical coherence tomography. We describe two rare cases of intrapapillary hemorrhage with adjacent peripapillary subretinal hemorrhage in an 11-year-old female and a 16-year-old male, both with no past history except myopia.

## Case presentation

### Case 1

**Fig. 1:**
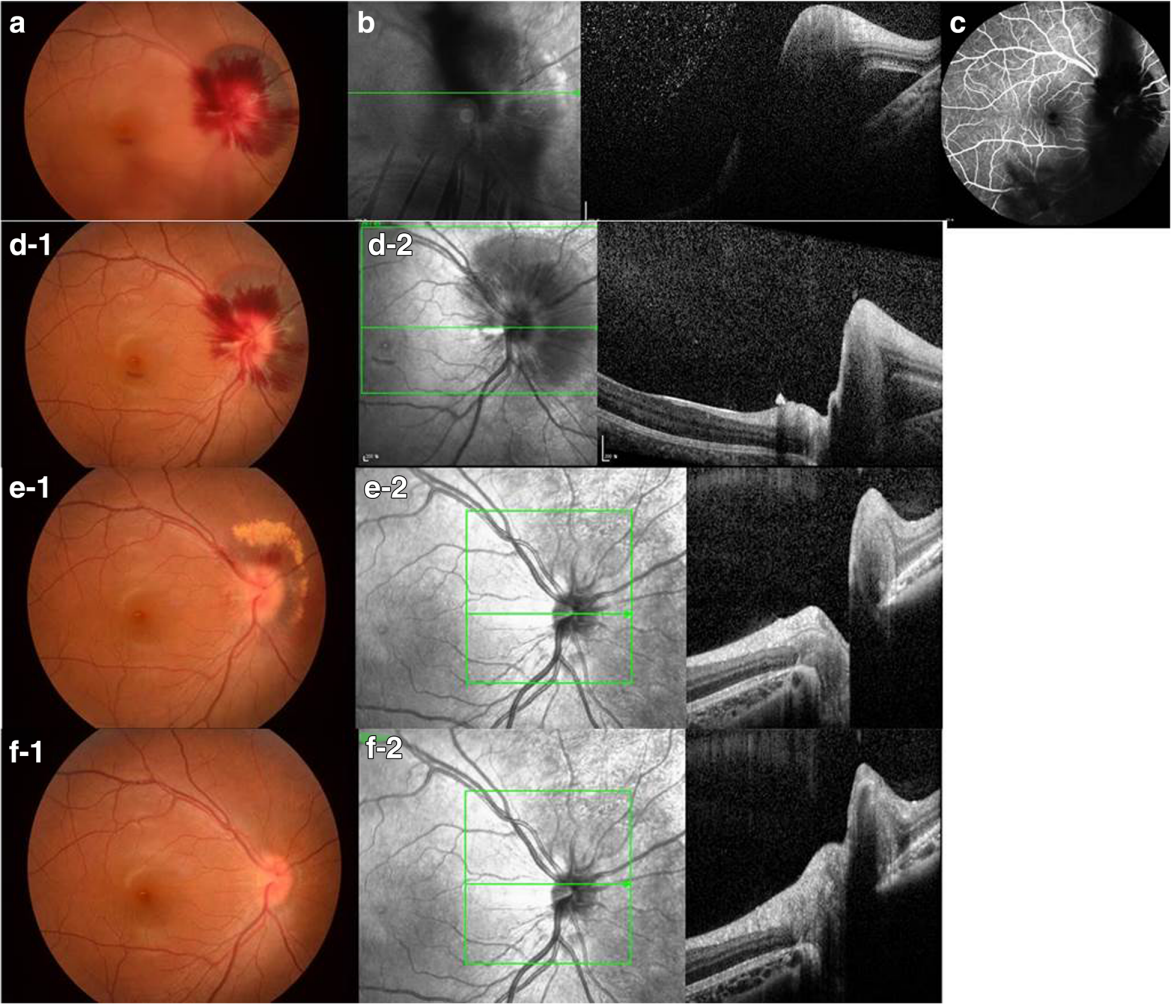
An 11-year-old female with no previous medical history presented with floater symptoms in her right eye. **a** Fundus examination showed disc hemorrhage with peripapillary subretinal hemorrhage in the affected eye. **b** Optical coherence tomography (OCT) revealed massive peripapillary subretinal hemorrhage. **c** Fluorescein angiography revealed blocked fluorescence due to peripapillary hemorrhage. Serial fundus photographs and OCT scans revealed spontaneous resolution of intrapapillary hemorrhage with adjacent peripapapillary subretinal hemorrhage at 1 week (**d**-1, 2), 4 weeks (**e**-1, 2), and 3 months (**f**-1, 2) after initial examination

**Fig. 2:**
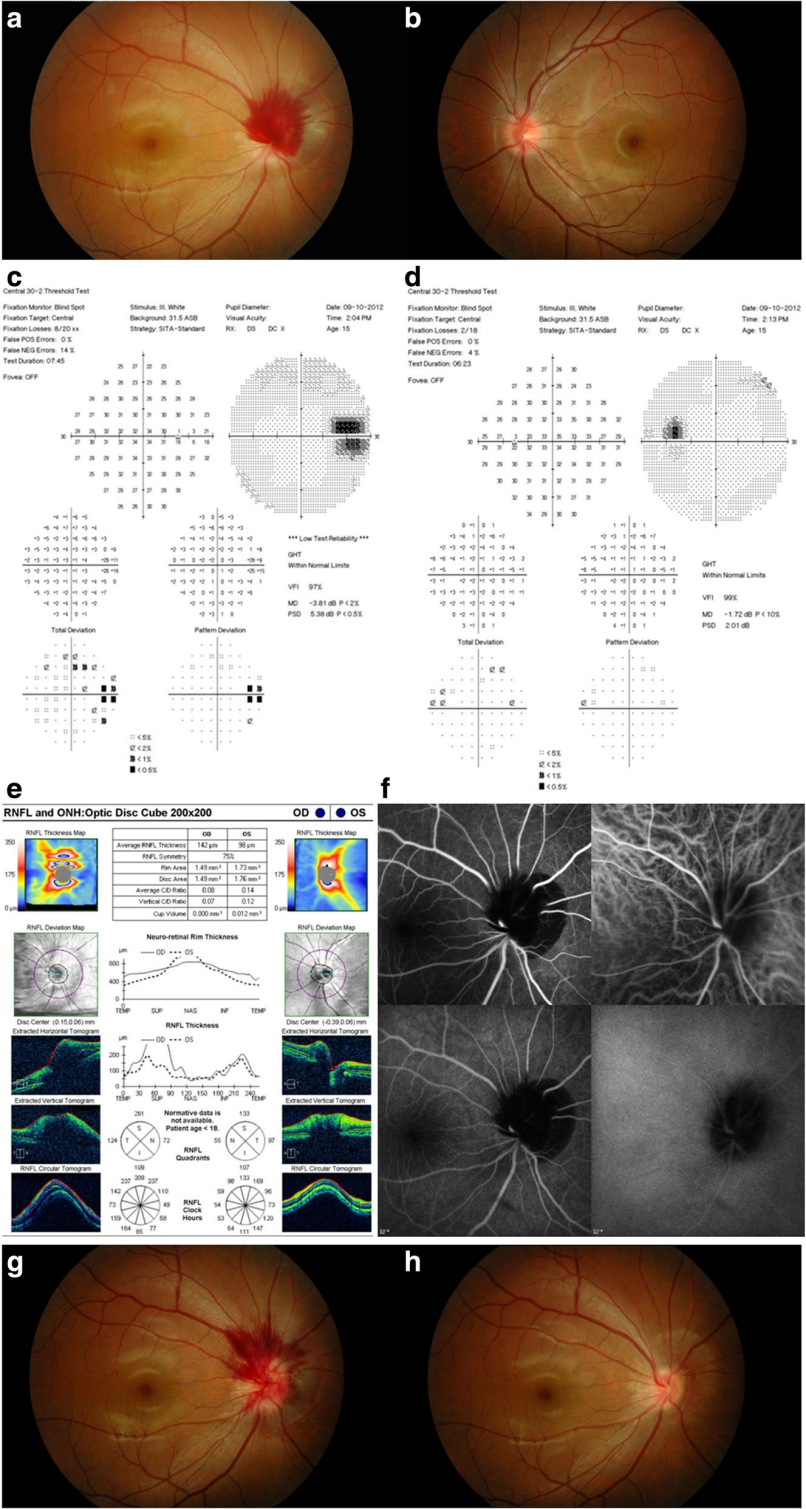
A 16-year-old male with no previous medical history presented with blurred vision and black floaters in his right eye. **a** Fundus examination showed intrapapillary hemorrhage with peripapillary subretinal hemorrhage on his right eye. **b** Fundus examination showed slight disc hyperemia on his left eye. **c** Visual field exams showed enlarged physiological scotoma in his right eye. **d** His left visual field exam was within normal limits. **e** Optical coherence tomography revealed disc swelling with hemorrhage. **f** Fluorescein and indocyanine green angiography showed fluorescein blockage due to massive intrapapillary hemorrhage. **g** After 2 weeks, fundus examination showed improved intrapapillary hemorrhage. **h** After 4 weeks, near resolution of the intrapapillary hemorrhage and peripapillary subretinal hemorrhage were noted in his right eye

An 11-year-old female with no previous medical history presented with floater symptoms in her right eye. She had no medical or ophthalmological history that may contribute to the condition, such as hypertension and thrombocytopenia, which can cause bleeding. The patient had no trauma or medication history. Her BCVA (best-corrected visual acuity) by the Snellen chart was 20/20 in both eyes, with intraocular pressure of 15 mmHg in her right eye and 16 mmHg in her left eye. Slit lamp examination revealed no specific findings in the anterior segment of both eyes and no relative afferent pupillary defect, but − 3.5 diopters of myopia was noted in both eyes. Fundus examination and optical coherence tomography showed tilted disc, intrapapillary hemorrhage, peripapillary subretinal hemorrhage, and mild vitreous hemorrhage in her right eye (Fig. 1a, b), Fluorescein angiography showed blocked fluorescence due to peripapillary subretinal hemorrhage at the early phase, but no definite leakage or new vessels were noted at the late phase (Fig. 1c). We recommended further evaluations, including brain magnetic resonance imaging (MRI), but the patient (with their guardian) chose to undergo only ophthalmological evaluation. After 4 weeks, the hemorrhage had partially resolved without any treatment (Fig. 1e-1, 2), and complete resolution was noted after 3 months, with a BCVA of 20/20 in her right eye (Fig. 1f-1, 2).

### Case 2

A 16-year-old male presented with symptoms of blurry vision and black filamentous floaters for 1 day. He had no previous medical history, and no trauma or medication history. His BCVA was 20/20 in both eyes, with intraocular pressure of 15 mmHg in his right eye and 19 mmHg in his left eye. The patient had − 7.0 diopter myopia in his right eye and − 7.5 diopter myopia in his left eye, with no definite relative afferent pupillary defect. Slit lamp examination showed no specific findings except mild vitreous hemorrhage in his right eye. Fundus examination showed intrapapillary hemorrhage and peripapillary subretinal hemorrhage in his right eye, and mild optic disc hyperemia in his left eye (Fig. 2a, b). Optical coherence tomography revealed peripapillary edema in his right eye (Fig. 2e). Fluorescein angiography showed blocked fluorescence because of peripapillary subretinal hemorrhage, but no fluorescence leakage or hyperfluorescence. (Fig. 2f) A Humphrey visual field examination of his right eye showed no specific sign except for enlarged physiological scotoma (Fig. 2c). No specific signs were noted in the brain and orbit MRI. After 4 weeks of observation, the intrapapillary hemorrhage and peripapillary subretinal hemorrhage subsided without any particular treatment, with a visual acuity of 20/20 (Fig. 2g, h).

## Discussion

Cases of intrapapillary hemorrhage with adjacent peripapillary subretinal hemorrhage are uncommon and have been reported only very rarely. It commonly presents (1) monocularly, (2) with higher prevalence in myopic eyes, (3) more often in eyes with tilted discs, (4) abruptly, (5) in young age, and (6) with good visual prognosis in the affected eye [1, 4]. Two cases of a 15-year-old male and 13-year-old female patient with myopic eyes have been previously reported in Korea in 2006 [3]. However, the exact cause was not known [1, 2, 4, 5]. Previous studies proposed potential pathogenic mechanisms including vitreopapillary traction, the hemorrhage of anatomically vulnerable prelaminar blood vessels in crowded optic discs, the hemodynamic effects of the Valsalva maneuver, and complications of optic disc edema [1, 4]. Prelaminar regions have an arterial blood supply from the peripapillary choroidal arteries and posterior short ciliary arteries, whereas the venous system of the prelaminar portion drains predominately to the central retinal vein with minor contributions to the peripapillary choroidal veins [6, 7]. The unique structure of the elevated superior and nasal margins of a tilted myopic disc might draw the retinal and choroidal tissues over and around the elevated edge. Therefore, the choroidal blood supply of the prelaminar optic nerve may lead to bleeding in patients with a tilted myopic disc, which may be spontaneous or triggered by an acute event. [8, 9]. Likewise, in our cases, the hemorrhage formed in young patients with myopic eyes, and the bleeding may have originated from capillaries of the peripapillary choriocapillaris and branches of the posterior ciliary artery that traverse the border tissue of Elschnig between the nasal side of the optic nerve and the adjacent inner aspect of the scleral canal [10].

In addition, the optic disc drusen can be the possible cause of subretinal hemorrhage as a result of direct mechanical compression. Superficial peripapillary vessels of small optic discs and scleral canals with optic disc drusen are vulnerable, and can be ruptured resulting in hemorrhages over the optic disc and peripapillary areas. [11–15] However, in our cases, multimodal imaging including OCT did not reveal any presence of optic disc drusen. Similarly in several previous studies, presence of any optic disc drusen was not detected in any of the cases with peripapillary subretinal hemorrhage [1–4].

## Conclusion

Intrapapillary hemorrhage with adjacent peripapillary subretinal hemorrhage occurs most commonly in young healthy patients with myopic eyes, but the resultant hemorrhages spontaneously resolve without any particular treatment and complications, with preserved visual acuity and optic nerve function. To provide patients with the most appropriate care and treatment, this unique disease entity should be differentiated from other causes of peripapillary subretinal and vitreous hemorrhage, such as polypoidal choroidal vasculopathy, optic neuritis, disc hemorrhage, optic disc drusen and other retinal and choroidal vascular diseases.

## Abbreviations

BCVA: Best-corrected visual acuity
MRI: Magnetic resonance imaging
OCT: Optical coherence tomography

## References

[1] Kokame GT, Yamamoto I, Kishi S, Tamura A, Drouilhet JH (2004). Intrapapillary hemorrhage with adjacent peripapillary subretinal hemorrhage. Ophthalmology.

[2] Kokame GT (1995). Intrapapillary, peripapillary, and vitreous hemorrhage. Ophthalmology.

[3] Kim DJ, Seo WM, Shin JP, Kim IT (2007). Two cases of intrapapillary hemorrhage with adjacent peripapillary subretinal hemorrhage. J Korean Ophthalmol Soc.

[4] Katz B, Hoyt WF (1995). Intrapapillary and peripapillary hemorrhage in young patients with incomplete posterior vitreous detachment. Signs of vitreopapillary traction. Ophthalmology.

[5] Cibis GW, Watzke RC, Chua J (1975). Retinal hemorrhages in posterior vitreous detachment. Am J Ophthalmol.

[6] Hayreh SS (2001). The blood supply of the optic nerve head and the evaluation of it - myth and reality. Prog Retin Eye Res.

[7] Onda E, Cioffi GA, Bacon DR, Van Buskirk EM (1995). Microvasculature of the human optic nerve. Am J Ophthalmol.

[8] Watanabe C, Shiraki J, Yoshinaga K (1981). Unilateral disc hemorrhage in young people [in Japanese]. Ophthalmology.

[9] Teng Y, Yu X, Teng Y, Xu B, Sun Q, Dong L, Su Y, Wu X, Dai B (2014). Evaluation of crowded optic nerve head and small scleral canal in intrapapillary hemorrhage with adjacent peripapillary subretinal hemorrhage. Graefes Arch Clin Exp Ophthalmol.

[10] Strouthidis NG, Yang H, Downs JC, Burgoyne CF (2009). Comparision of clinical and three-dimensional histomorphometric optic disc margin anatomy. Invest Ophthalmol Vis Sci.

[11] Sanders TE, Gay AJ, Newman M (1970). Drusen of the optic disk-hemorrhagic complications. Trans Am Ophthalmol Soc.

[12] Rubinstein K, Ali M (1982). Retinal complications of optic disc drusen. Br J Ophthalmol.

[13] Romero J, Sowka J, Shechtman D (2008). Hemorrhagic complications of optic disc drusen and available treatment options. Optometry.

[14] Dhingra N, Prasad S (2003). Optic disc drusen. J Postgrad Med.

[15] Lee KM, Hwang JM, Woo SJ (2014). Hemorrhagic complications of optic nerve head drusen on spectral domain optical coherence tomography. Retina.

